# Dedication increases productivity: an analysis of the implementation of a dedicated medical team in the emergency department

**DOI:** 10.1186/s12245-017-0136-9

**Published:** 2017-02-21

**Authors:** Pedro Ramos, José Artur Paiva

**Affiliations:** 1 Faculty of Medicine, University of Porto, Porto, Portugal; 2 Medical Director Office, Hospital das Clínicas da Faculdade de Medicina da Universidade de São Paulo, São Paulo, Brazil; 3 Autonomous Management Unit of Emergency and Intensive Care Medicine of Centro Hospitalar São João, Porto, Portugal

**Keywords:** Doctors, Hospitals, Healthcare team, Organisation efficiency

## Abstract

**Background:**

In several European countries, emergency departments (EDs) now employ a dedicated team of full-time emergency medicine (EM) physicians, with a distinct leadership and bed-side emergency training, in all similar to other hospital departments. In Portugal, however, there are still two very different models for staffing EDs: a *classic* model, where EDs are mostly staffed with young inexperienced physicians from different medical departments who take turns in the ED in 12-h shifts and a *dedicated* model, recently implemented in some hospitals, where the ED is staffed by a team of doctors with specific medical competencies in emergency medicine that work full-time in the ED.

Our study assesses the effect of an intervention in a large academic hospital ED in Portugal in 2002, and it is the first to test the hypothesis that implementing a dedicated team of doctors with EM expertise increases the productivity and reduces costs in the ED, maintaining the quality of care provided to patients.

**Methods:**

A pre–post design was used for comparing the change on the organisational model of delivering care in our medical ED. All emergency medical admissions were tracked in 2002 (classic model with 12-h shift in the ED) and 2005/2006 (dedicated team with full-time EM physicians), and productivity, costs with medical human resources and quality of care measures were compared.

**Results:**

We found that medical productivity (number of patients treated per hour of medical work) increased dramatically after the creation of the dedicated team (X^2^_*KW*_ = 31.135; *N* = 36; *p* < 0.001) and costs with ED medical work reduced both in regular hours and overtime. Moreover, hospitalisation rates decreased and the length of stay in the ED increased significantly after the creation of the dedicated team.

**Conclusions:**

Implementing a dedicated team of doctors increased the medical productivity and reduced costs in our ED. Our findings have straightforward implication for Portuguese policymakers aiming at reducing hospital costs while coping with increased ED demand.

## Background

For several decades, European emergency departments (ED) have been handled by junior doctors from different specialties with no specific training in emergency care, who took weekly shifts in the ED and soon returned to their original departments [1]. This meant that quality improvement strategies within the ED setting were usually compromised: responsibility for the emergent patient was regularly fragmented, case discussion was fragile, work environment was hostile and management strategies were difficult to implement [2, 3]. With increased understanding of the mission of the ED within the hospital and the health system, emergency care has gained *momentum* in Europe: emergency medicine (EM) is now a recognised medical specialty in several countries including Italy, The Netherlands, Sweden and Finland [4–8], and in some of these countries, EDs now employ a dedicated team of full-time EM physicians, with a distinct leadership and bed-side emergency training, in all similar to other hospital departments [6, 9].

In Portugal, these changes have been slower. Since 2002, EM is considered an “area of competence”, but not a medical specialty. Moreover, most Portuguese EDs still operate in the “classic” medical system, with junior physicians from different specialties working on a part-time basis in the ED and some senior internal medicine and/or general surgery doctors supervising the ED, but few actually assuming its leadership [10, 11]. Furthermore, the lack of organisation within these EDs often led to an increase in costs by an accumulation of overtime work [12].

Nonetheless, inspired by the model of ED care in other European countries, some Portuguese hospitals have not waited for government decisions and started to change their internal organization: they defined the ED as a department of its own within the hospital hierarchy, appointed an ED medical director with EM experience and created a full-time dedicated team for handling emergency care [13, 14]. Considering both the stressful nature of ED work and the uncertainty surrounding the future of EM career in Portugal, these teams were offered higher salaries compared to typical ED hourly wages in Portugal. If the model is cost-effective, higher productivity resulting from having a team fully dedicated to the ED should outweigh the costs of setting up this dedicated team.

Our study uses data from a large academic hospital in Portugal to test the hypothesis that implementing a dedicated team of doctors with EM expertise increases the productivity and reduces costs in the ED, maintaining the quality of care provided to patients.

Even though our study assesses an intervention with more than a decade old, when the dedicated medical team was created in our ED, to our knowledge, we are the firsts to evaluate the impact of a dedicated medical team model in terms of productivity and costs. Therefore, the implications of our work may be important considering the economic crisis and financial restrictions occurring in several European countries, namely in Portugal, and for the ongoing development of emergency medicine in Europe.

### The Portuguese context

The Portuguese Emergency Network comprises three types of EDs—small and often rural “basic” EDs, intermediate “medical–surgical” EDs and urban “polyvalent” EDs—which differ in terms of the complexity of the cases received, the resources available (e.g. human resources, lab and imaging exams) and the price the government pays the hospitals for each ED visit.

Polyvalent emergency services usually serve a population of 750,000–1,000,000 people and are required to have specific medical expertise in order to receive the most complex patients (e.g. neurosurgery, cardiothoracic surgery and vascular surgery medical specialists; Digital Angiography and MRI available; Pre-defined emergency clinical pathways for chest pain, stroke, major trauma and sepsis, …). Basic emergency departments are small proximity services that only receive simple cases, but need to have the skills for stabilising and transferring trauma patients for higher-level EDs. Medical–surgical emergency departments are at an intermediate level, referring the most complex patients to polyvalent emergency services and receiving some cases from basic EDs. Therefore, they are required to have a 24-h staffed emergency OR and ED imaging with CT and ultrasonography available [15].

More importantly in the context of this study, there are two very different medical staffing frameworks across Portuguese EDs: a *classic* model, where doctors from different medical departments take turns in the ED in 12-h shifts and a *dedicated* model, recently implemented in some hospitals, where the ED is staffed by a team of doctors with specific medical competences in emergency medicine that work full-time in the ED. Table 1 presents the key features for each of these models for staffing EDs in Portugal.

**Table 1:** Key features for the two type of models of medical staffing of EDs in Portugal

Classic model	Dedicated model
The ED is handled by doctors from different medical specialties (primarily junior doctors in training)	There is a team of doctors with formal training in emergency medicine (primarily consultants/senior doctors)
Part-time—12 to 18 h/week in the ED	Full-time—40 h/week in the ED
The ED director has no direct leadership responsibilities over the medical staff (i.e. each medical doctor answers to his department head)	The ED direct has a formal leadership role over the medical team
Inexistent ED recruiting policies (staffing is dependent on other departments’ needs)	There is an active recruitment based on doctors’ vocation for the ED work
The ED’s strategy and leadership structure is unclear to most doctors that occasionally work in the ED, and the medical staff is usually not aware of key performance indicators in the ED	The ED is a hospital department on its own, with a leadership structure, a clear strategy aligned to the hospital’s mission and vision and a regular monitoring and discussion of key performance indicators in the ED
Scarce training in the ED (e.g. advanced life support, trauma patient, …)	Formal training courses in the ED on a regular basis
Doctors who occasionally visit the ED for patient care are less committed to quality improvement measures, case discussion, clinical audit activities, …	Doctors from the team, who continuously work in the ED, are more committed to quality improvement measures, process engineering, case discussion, clinical audit activities, team-building initiatives, …

Source: National Report of the Commission for the Emergency Department Network Reform [15]

## Methods

### Setting

*Centro Hospitalar São João (CHSJ)* is a tertiary academic hospital that serves a population of 750,000 in the North of Portugal. Its ED, classified as a polyvalent ED, has an average volume of 150,000 annual visits and an internal organisation based on a dedicated team of doctors with EM experience, set up in 2005–2006.

In 2002, the ED was based on the “classic model” of medical staffing, where emergency care was handled by doctors from several medical specialities, who took turns in the ED, in 12-h shifts (doctors up to the age of 55^1^ had a specific weekly 12-h shift in the ED), returning to their activities in their original department during the rest of the week.

In 2005, an organisational change was performed in the ED: medical (but not surgical) emergency care became handled by a dedicated team of ED doctors, who occasionally ask for specialised consultancy from other doctors of selected medical specialities. In these specialties, doctors are assigned to their regular department duties (outpatient care, inpatient admissions, residents’ and medical students’ training,…), but each department has to appoint one or more on-call consultant doctors that may be contacted by the ED to examine specific patients.

Since a formal emergency medicine speciality is not established in Portugal, the doctors that took part in this team had different medical backgrounds, but participated in a specific training program organised by the ED’s leadership staff. This training program provided competence in some basic areas—advanced life support, trauma, intensive medicine, sepsis, resuscitation ….—and consisted of multiple compulsory courses throughout the year, namely in *electrocardiography*, *ED imaging*, *neurologic and cardiologic emergencies*, *critically ill patient transfer*, *acute respiratory failure and non-invasive ventilation*, *infection and sepsis*, *disaster medicine and medical contingency planning*. The dedicated team was also responsible for a newly created module in the medical school curriculum—Intensive Medicine and Emergency Care—which included an advanced life support course, ED clinical case discussion and ED work in the emergency room and EM teams for medical students interested in this area.

On top of the EM training provided, the dedicated team was responsible for creating clinical protocols for the interface between the ED and other medical services in the hospital (e.g. hospitalisation criteria, lab and imaging protocols) and for rolling-out some key clinical protocols within the ED, jointly with regional and national public health institutions. Specifically, four emergency clinical pathways were implemented during this period (stroke, chest pain, trauma and sepsis) that are now also widely used in other Portuguese EDs. These are clinical algorithms for evaluating and treating these diseases, based on the understanding that time for treatment is key for the achieved clinical outcome “time is tissue”. The acute stroke care pathway, for instance, implemented in 2005, focused on creating an evidence-based systematic approach to stroke patients, by identifying stroke symptoms using the National Institute of Health Stroke Scale (NIH-SS), considering inclusion/exclusion criteria for thrombolysis and quickly referring patients with suspected acute stroke to the stroke unit, if appropriate.

### Study design

A *pre–post* design was used for comparing these two organisational models of delivering emergency care in CHSJ’s medical ED, by estimating the productivity variation between 2002 (classic model) and 2005/2006 (dedicated model). We chose to study the dedicated model in 2005 and in 2006,^2^ rather than in more recent years, in order to make costs and outcomes more comparable with 2002, a strategy that has been chosen in similar studies [16]. Note, for instance, that there were several changes in the last 10 years in the Portuguese National Health Service that may have had an effect on ED demand (e.g. reforms in our primary health care sector, increase in co-payments for ED visits,…) and supply (e.g. reorganisation in the ED network that closed some small EDs; the economic crisis reduced medical staff’s wages; …). Reporting outcomes after these policies occurred could arguably bias our results, by incorporating effects that were unrelated with our intervention within the ED.

Moreover, we analysed ED productivity separately for 2005 and 2006, since in 2005 the dedicated model was only partially implemented, i.e. there were still some few doctors working in the classic “shift” basis.

Productivity was measured in the conventional management way: ED visits (ED’s production) divided by the hours of medical work used in that production.

In order to isolate only the “medical” work (and not the “surgical” work^3^) in the ED, we selected ED visits whose discharge was the responsibility of doctors from departments of medical specialities.^4^

For identifying the hours of medical work devoted to treating these patients:

- In 2002 (classic model), we included the 12-h shifts made by doctors who were assigned to the ED by law (doctors up to the age of 55).
- In 2005 and 2006 (dedicated model), we included the work hours of doctors who were part of the dedicated team, as well as the hours of medical specialists outside the team who were called to the ED for consultancy on specific cases (consultancy hours). Specifically for junior doctors in the first 2 years of residency, their training requires 12 h/week of work in the ED during these years (i.e. these are junior doctors who assist the dedicated team on a daily basis), whereas other junior doctors who are senior residents do not work in the ED on a regular basis, but are often called to the ED for consultancy on specific cases (consultancy hours).
- In each year, we also include overtime and (home) on-call specialist coverage that were made by medical doctors in the ED.

In addition to productivity analysis, we also studied the costs of both models. For this purpose, we used the gross remuneration^5^ earned by these doctors, with a methodology that overlaps the one used to account for the hours of work—a 12-h proportional salary in 2002 and the full-time salary in 2005 and 2006.

In addition to this productivity and cost analysis, we also analysed the impact of the implementation of this dedicated team on the ED’s quality of care. For doing so, we identified a set of ED quality indicators that are widely used in national [15] and international studies [17]. These include total duration of the ED visit (LOS), readmission rate to the ED within 24, 48 and 72 h, hospitalisation rate, left without being seen by a doctor (LWBS) rate and in-ED mortality rate.

Kruskal–Wallis test and Mann–Whitney *U* test were used to make comparisons between years and *t* tests for monthly data. All statistical analyses were made with STATA12®.

## Results

### Demographic characteristics

We identified a set of 153,300 visits in the ED with discharges made by doctors of medical specialities, throughout the period of our analysis. Some demographic characteristics of our ED adult patients are presented in Table 2, which shows that baseline characteristics of our ED population have not changed during this period.

**Table 2:** ED patients’ demographic characteristics

Demographic information	2002	2005	2006
Number of women (%)	21,520 (50.7%)	26,983 (51.5%)	31,206 (53.0%)
Age (average ±SD)	52 ± 21	54 ± 21	53 ± 21
Distance (average ±SD) (km)	17.1 ± 26.1	15.5 ± 26.7	15.3 ± 25.2
Patients with NHS insurance (%)	37,262 (88.9%)	46,327 (88.4%)	42,227 (89.1%)
Patients with public employees’ insurance (%)	3058 (7.3%)	3897 (7.4%)	3344 (7.1%)

### Production of the ED

In the period after the implementation of the dedicated team, there was an increase in the number of ED visits of 25% in 2005 and 40% in 2006. This increase occurred with a continuous reduction of the work carried out by doctors from medical departments (e.g. cardiology or neurology medical specialists), with a progressive uptake by the ED dedicated team. Table 3 also shows that, after the intervention, the majority of ED patients were treated by senior doctors, who were part of the dedicated full-time team in the ED, while in 2002 they were primarily handled by junior doctors, who worked part-time in the ED.

**Table 3:** ED medical discharges by doctors’ career category

Career category	2002	2005	2006
Department head	430 (1.0%)	464 (0.9%)	196 (0.3%)
Senior doctor	16,781 (40.0%)	40,464 (77.3%)	45,766 (77.6%)
Junior doctor	24,108 (57.6%)	9962 (19.0%)	11,585 (19.6%)
Self-employed doctor	590 (1.4%)	1499 (2.9%)	1455 (2.5%)

Senior doctors are consultants who have completed their specialist training. Junior doctors are medical doctors who are still in a residency training position in their chosen medical speciality. “Self-employed” doctors are medical specialists who are not part of the permanent hospital workforce and are usually employed during peak periods

Table 4 shows that in the years after the intervention there was an increase in the average ED visit duration (time in the ED) and in the LWBS rate and an increase in readmission rates in 2006. In contrast, the hospitalisation rate significantly decreased after the intervention.

**Table 4:** Quality indicators of ED’s production; *p* value based on *t* tests comparing performance indicators between 2002 and 2005 and 2002 and 2006

Production in the ED	2002	2005	*p* value	2006	*p* value
Av. monthly visits	3493	4366	<0.001	4917	<0.001
Quality indicators					
Hospitalisation rate	24.7%	19.1%	<0.001	17.3%	<0.001
Total time in the ED (average ± sd)	04:34:00 ± 06:04:20	05:48:34 ± 04:56:59	<0.001	05:50:25 ± 04:55:29	<0.001
Readmission rate at 24 h	1.1%	1.0%	0.53	1.3%	<0.001
Readmission rate at 48 h	2.1%	2.0%	0.28	2.4%	<0.001
Readmission rate at 72 h	2.9%	2.7%	0.14	3.3%	<0.001
ED mortality rate	0.4%	0.6%	<0.001	0.5%	0.07
LWBS rate	0.9%	1.4%	<0.001	4.5%	<0.001

### Hours of medical work

**Fig. 1:**
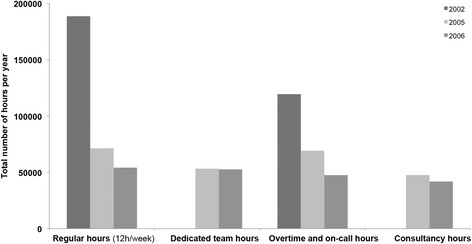
Evolution of the work hours by category. Both regular work hours and overtime decreased significantly between the classic model and the dedicated model. Work hours in every category also decreased between 2005 (dedicated model in adjustment) and 2006 (dedicated model)

In addition to the increase in the number of patients treated, we found a pronounced decrease in the total hours of work, of 22% between 2002 and 2005 and of 36% between 2002 and 2006. Moreover, between 2005 and 2006, there was still a reduction of nearly 19% in the total hours of work. Figure 1 presents this evolution of the work hours in the ED, subdividing them by category. It shows that the needs in both regular hours and overtime were significantly higher in 2002 compared to following years.

With the information on the number of patients treated and the hours of work, we calculated ED productivities, in each year (Table 5).

**Table 5:** Productivity and costs in the ED in 2002, 2005 and 2006

	2002	2005	2006
Productivity (patients treated/hour)	0.13	0.20	0.27
Productivity variation		53.80%	107.69%
Cost with ED’s medical hour (in 2015 values)	6,544,622€	4,695,463€	3,602,738€
Cost variation		−28.25%	−44.95%
Hour cost per patient visit (in 2015 values)	165.48€	98.20€	67.65€

There were statistically significant differences, using the Kruskal–Wallis test, between the productivity of the several years (X^2^_*KW*_ = 31.135; *N* = 36; *p* < 0.001). Those differences were shown to be significant both between 2002 and 2005 and between 2005 and 2006, using the Mann–Whitney *U* test.

### Costs with the work in the ED

The bottom part of Table 5 presents the evolution in the cost of the medical HR throughout the period of our analysis. There was a very pronounced cost reduction between 2002 and 2005 and 2006, yet with lower magnitude compared to the productivity increase described above.

### The ED quality of care in following years

**Fig. 2:**
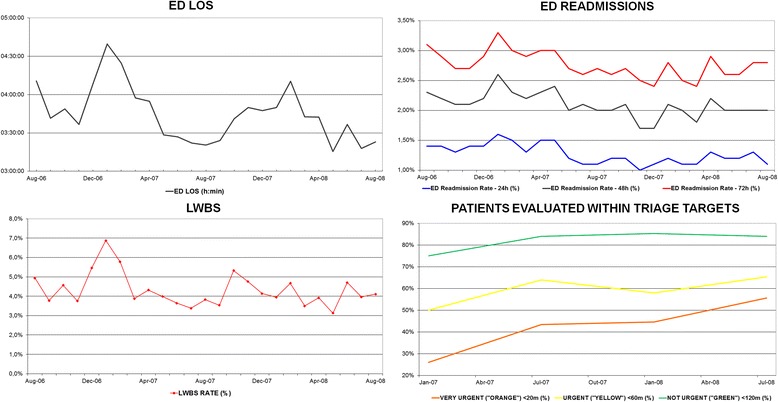
Evolution of key ED quality indicators in the years after the implementation of the dedicated medical team in the ED

In the last section, we provided evidence that the implementation of a medical team of doctors working full-time in the ED had noteworthy effects on the productivity and HR costs in our ED. One concern that may remain is whether these improvements in productivity had detrimental effects in the quality of ED care in the years after the creation of the team. In Fig. 2, we provide the evolution of some key ED quality indicators in the period after the implementation of the dedicated ED team. Figure 2 shows that ED LOS, readmission rates and LWBS rates decreased considerably, whereas the proportion of patients seen by a physician within the time recommended at triage increased continuously in the 2 years after our intervention.

## Discussion

Our study shows an increase in productivity and a decrease in medical HR costs with the creation of a dedicated team in the ED of an academic urban hospital. Some factors that contributed to these pronounced differences in productivity were not only an increase in the number of patients treated, but mainly a reduction in the necessary number of work hours for treating those patients, achieved both in regular work hours and in overtime hours.

There is a large literature documenting that a dedicated team of senior doctors is associated with higher productivities in the ED. Sen et al. and Christmas et al. found that increasing senior doctors’ coverage during nights in the UK led to more patients being treated per hour [18, 19]. Austin et al. also reported an increase in the volume of patients in the ED following the creation of a dedicated surgical team [20].

Similarly to these authors, we argue that this organisational change may improve productivity by enhancing some of the practices that have been signalised as high performance management practices [21]. Morey et al. found that the implementation of formal teamwork structures had positive effects on staff attitudes and team behaviours in the ED [22]. Salas et al. described core principles for effective teamwork in emergency medicine [23]: existence of identifiable leadership and shared understanding of the tasks, purposes and goals for the team, implementation of training and feedback/“learning from mistakes” practices and development of appropriate team cooperation and communication skills. Some—if not all—of these practices are hallmarks of this transition from “classic model” to “dedicated model” of delivering care in our ED and may help to explain the organisational benefits we have found.

Moreover, the implementation of the dedicated team directly resulted in increased physician seniority in the hospital’s ED. There is compelling evidence that increasing the number of senior doctors in the ED improves the quality of care and reduces costs in the ED [24, 25].

Consistent with the increase in productivity, we also found a reduction of the costs of ED medical work between 2002 and 2005 and 2006. However, this reduction was lower than the productivity variation, due to the intrinsic cost of setting up the dedicated team (*setup costs*). One important setup cost with the dedicated model arose from contracting doctors under an individual employment contract, rather than under civil servant status, which allowed offering higher remunerations, a strategy that has also been employed in other settings [19]. This was particularly important in our context considering the nature of ED work and the non-existence of a medical speciality of emergency medicine in Portugal.

We also evaluated the quality of ED care delivery in each year. We found a very significant decrease in the hospitalisation rate and an increase in the average length of stay within the ED. We believe these to be direct consequences of this new model of delivering ED care, i.e. the formalisation of hospitalisation criteria and laboratory and imaging protocols for the ED patient increased ED patients’ length of stay while reducing unnecessary hospitalizations (e.g. patients that were hospitalised for a short-stay reassessment or lab results and/or waiting for stabilisation of their clinical condition). Reductions in admission rates from the ED have also been found in other studies that analysed the effect of increasing senior doctors’ presence in the ED [18, 19].

We also found an increase in the LWBS and readmission rates in 2005 and 2006. Nonetheless, in a comparative study about the implementation of the Manchester Triage System in several Portuguese EDs, our LWBS and readmission rates are similar to other tertiary EDs that have implemented the triage system [26]. Therefore, we hypothesise that, at least for the LWBS, this increase may be due to the formalisation of the Manchester Triage in the ED during the period of implementation of the dedicated team, which may have led to an increase in drop-outs in the less severe visits, which account for a high share of the ED’s volume [15, 27].

Mortality rates have also increased in 2005. One possible explanation for this finding is the influenza epidemic that hit Portugal in the first months of that year [28].

Nevertheless, our study has some limitations. The most significant arises from our need to use data from 2002 to 2006, when the intervention took place in our hospital. Although HR medical costs should not have changed in the last years, since salaries in the public sector are regulated by national pay scales, there may still be some doubts as to whether the cost reduction we report would still be found nowadays. In a study using more recent data, Oliveira (2012) simulated the economic impact of the hypothetic creation of a dedicated team in another Polyvalent ED that is close to the same size and in the same region of ours [29]. The author estimated a predicted cost reduction of 30% in medical HR costs if they implemented a dedicated team in their ED, which is similar to the one we present in this study. This reassures us of the robustness of our findings.

Our study also lacked data on the number of exams and prescribed medication, which may have also changed with the implementation of a senior dedicated team. Further studies may try to include these costs and compare them across the two models.

## Conclusions

In conclusion, there are some clear practical implications of our findings. Several hospitals in Portugal face pressure for introducing cost containment measures, yet demand for ED is increasing and cyclically threatens the health system. We show that changing the organisational model in the ED by creating full-time dedicated medical teams of doctors with EM expertise may have noteworthy financial effects, by releasing doctors from their ED duties, reducing overtime work costs and avoiding over allocation of HR elsewhere in the hospital.

## Footnotes

1 Doctors older than 55 have a leave of absence from ED work.

2 We used 2005 and 2006 as our post-intervention period. 2003–2004 was a transition period when some few organisational measures were already occurring (e.g. appointing a dedicated medical coordinator in the ED) but the full dedicated team had not still been created.

3 Note that the surgical work in the ED (e.g. general surgery and orthopaedics) was not targeted by this intervention.

4 We have only considered doctors from medical departments—internal medicine, cardiology, neurology, dermatology, gastroenterology, pulmonology, infectious diseases, rheumatology nephrology, endocrinology, immunoallergology and hemato-oncology. We have also included the doctors assigned to the departments of the intensive care unit and anaesthesiology, because they were assigned to the emergency room.

5 These remunerations were adjusted to the inflation rate reported by *Statistics Portugal*.
